# Research on dynamic functional connectivity and graph theoretic features of different motor subtypes in Parkinson’s disease based on ICA

**DOI:** 10.3389/fneur.2026.1943343

**Published:** 2026-10-08

**Authors:** Jia Tang, Shaoge Gao, Yichen Lv, Hao Wang, Yanting Liu, Hongyu Zhou, Jiaqi Yuan, Xueqian Lin, Manyan Hu, Yan Cong, Xin Wang

**Affiliations:** 1 Department of Neurology, Yangzhou Traditional Chinese Medicine Hospital Affiliated to Nanjing University of Chinese Medicine, Yangzhou, China; 2 Department of Neurology, Taizhou Fourth People's Hospital, Taizhou, China; 3 Department of Rehabilitation Medicine, Northern Jiangsu People's Hospital, Yangzhou, China; 4 Department of Rehabilitation Medicine, Yiwu Maternity and Children Hospital, Yiwu, China

**Keywords:** brain functional networks, dynamic functional connectivity, dynamic graph theory analysis, independent component analysis, motor subtypes, Parkinson’s disease, resting-state functional magnetic resonance imaging

## Abstract

**Objective:**

This study combined independent component analysis (ICA)-derived dynamic functional connectivity (dFNC) with dynamic graph theory analysis methods to analyze the changes in dFNC and dynamic graph theoretic indicators in different motor subtypes of Parkinson’s disease (PD)—tremor-dominant (TD) subtype and postural instability/gait difficulty (PIGD) subtype—and healthy controls (HC). We aimed to explore the neural circuitry abnormal mechanisms and differences between the two subtypes.

**Methods:**

Using a prospective observational study design, from January 2021 to May 2024 at Northern Jiangsu People’s Hospital, we enrolled 36 diagnosed PD patients according to inclusion criteria—19 patients with PIGD subtype and 17 patients with TD subtype—along with 19 healthy subjects matched for age, sex, and education level. Resting-state functional magnetic resonance imaging (rs-fMRI), demographic information, and clinical motor function assessment data were collected from all participants. The ICA and dynamic functional network connectivity (dFNC) analysis were performed using the GIFT toolbox platform based on MATLAB. Combined with dynamic graph theory analysis, features such as characteristic path length, clustering coefficient, and node degree were extracted. The dFNC and dynamic graph theoretic indicators of the PIGD group, TD group, and HC group were compared horizontally to clarify the common and specific dynamic abnormalities of brain networks between the two subtypes.

**Results:**

Nine functional brain networks were identified through ICA. Significant differences among the three groups were observed in five brain networks: auditory network, left frontoparietal network, right frontoparietal network, ventral attention network, and sensorimotor network. Based on network-to-network functional connectivity analysis using these nine functional brain networks, after strict FDR correction, only inter-network coupling strength between the auditory network and sensorimotor network showed significant group differences (*T* = 7.548, FDR-corrected *p* = 0.00137). Four recurring functional connectivity states were identified through dFNC analysis. Across all participants, State 2 and State 3 accounted for 38 and 23% of total time, respectively. PIGD patients tended to spend more time in these two low-connectivity states. In terms of temporal properties, only the occurrence frequency of state 4 showed significant differences among the three groups (*F* = 4.855, FDR-corrected *p* = 0.047). Post-hoc test results showed that HC groups were significantly higher than PIGD groups and presented a trend higher than TD groups, while there was no statistically significant difference between the two PD subtypes. One-way analysis of variance was performed on whole-brain node-to-node functional connectivity and graph theoretic attributes of the three groups; no significant differences were found after FDR correction.

**Conclusion:**

Compared with the HC group, PD patients (both TD and PIGD groups) exhibited spatial distribution abnormalities in multiple brain networks. Nominally significant alterations suggestive of compensatory activation of the motor cortex were observed at the uncorrected statistical level, alongside weakened functional connectivity in specific networks.

## 1 Introduction

Parkinson’s disease (PD) is the second most common neurodegenerative disease worldwide, following Alzheimer’s disease (AD). Biologically, its main pathological characteristics include progressive loss of substantia nigra-striatal neurons and intracellular Lewy body aggregation. Notably, clinical symptoms do not manifest until approximately 50–60% of corresponding neuronal cells have died (1). Some studies (2) indicate that PD can typically be diagnosed when 60% of substantia nigra-striatal neurons and 80% of dopaminergic neurons in the striatum are lost, highlighting the urgent need for early diagnosis of PD. Resting-state functional magnetic resonance imaging (rs-fMRI) has become a commonly used tool for researching PD, as it can detect abnormal changes in brain functional activity and functional connectivity (FC) in PD patients (3, 4). This study focuses specifically on the postural instability/gait difficulty (PIGD) subtype and tremor-dominant (TD) subtype of PD, employing independent component analysis (ICA) (5)—a data-driven, statistical method that decomposes observed multidimensional signals into mutually statistically independent components by exploiting their independence and non-Gaussianity—as the core analytical method to decompose blood oxygen level-dependent (BOLD) signals from all brain voxels into spatially and temporally independent signals, directly measuring interactions within and between multiple brain networks, thereby grouping brain regions with higher correlation levels into functional brain network areas (6). On this basis, we combined dynamic functional network connectivity (dFNC) analysis methods based on ICA to calculate differences in dynamic connection strength over time between different independent brain networks and their temporal evolution features (7); additionally, we introduced dynamic graph theory methods to model brain networks using graph structures (with brain regions as nodes and neural connections as edges), calculating dynamic graph theoretic indicators such as characteristic path length, clustering coefficient, and node degree, further quantifying the dynamic topological structure characteristics of PIGD subtype, TD subtype, and healthy control (HC) group brain networks, and comprehensively elucidating the dynamic change patterns of brain functional networks in different PD subtypes.

PD patients with different motor subtypes exhibit significant heterogeneity in motor symptoms, cognitive function, and non-motor symptoms. Although their pathological basis centers on damage to the nigrostriatal pathway, motor control and cognitive regulation in the body are not accomplished by a single brain region alone; rather, they depend on the collaborative coordination of multiple brain networks, including the motor network, default mode network, and frontoparietal executive network (8). Although both subtypes share nigrostriatal pathway damage as their pathological basis, motor and cognitive control rely on the coordinated interaction of multiple brain networks. Notably, the two subtypes differ markedly in clinical symptom profiles—TD patients present predominantly with resting tremor, whereas PIGD patients exhibit more severe axial symptoms, bradykinesia, cognitive impairment and faster disease progression. These phenotypic differences suggest that the underlying neural circuit involvement—and hence the pattern of functional connectivity among the corresponding brain networks—may differ between the two subtypes. Accordingly, we hypothesized that TD and PIGD subtypes would exhibit subtype-specific alterations in functional connectivity and topological organization, which could be captured by dynamic connectivity and graph-theoretic analyses.

We chose ICA because it can decompose whole-brain BOLD signals into spatially independent components corresponding to distinct functional networks, allowing direct measurement of interactions within and between networks. ICA-based dFNC has been widely applied in resting-state fMRI studies and has successfully revealed time-varying connectivity states in both healthy populations and patients with neuropsychiatric and neurodegenerative disorders (9, 10), underscoring its utility for characterizing dynamic brain network alterations. In parallel, graph-theoretic analysis is an established framework for quantifying brain network topological properties across neurological diseases (11). Combining ICA-derived dFNC with dynamic graph metrics therefore offers a more comprehensive, complementary view of both temporal dynamics and topological structure than either static connectivity or conventional single-metric analyses alone.

Given this background, do PIGD subtype and TD subtype exhibit specific differences in connection patterns and topological structures of brain functional networks? Are these differences closely related to their specific clinical symptom profiles? This study aims to explore this in depth through neuroimaging approaches, providing theoretical foundations for precise subtyping and mechanistic research of different PD subtypes.

## 2 Methods

### 2.1 General information

Patients with PD diagnosed at Northern Jiangsu People’s Hospital from January 2021 to May 2024 were recruited, along with healthy subjects matched for age, sex, and education level. This study was approved by the Ethics Committee of Northern Jiangsu People’s Hospital and registered through the Chinese Clinical Trial Registry (registration number: ChiCTR2000041356). All subjects signed informed consent forms prior to enrollment.

### 2.2 Case selection

Inclusion criteria for PD patients: (1) Diagnosed with primary PD according to the Chinese Diagnostic Criteria for PD (2016 edition) (12); (2) All were right-handed.

Exclusion criteria: (1) Patients with Parkinson’s plus syndromes such as multiple system atrophy or progressive supranuclear palsy; (2) Patients with history of intracranial hemorrhage, cerebral infarction, head trauma, tumors, or neurosurgery; and (3) Patients with contraindications for MRI scanning. All PD patients underwent MRI scanning and clinical scale assessment after at least 12 h of discontinuation of anti-PD medications. Clinical assessment scales included: Unified Parkinson’s Disease Rating Scale Part III (UPDRS-III), Hoehn-Yahr staging, Mini-Mental State Examination (MMSE), and Montreal Cognitive Assessment (MoCA).

### 2.3 PD patient grouping criteria

According to the method proposed by Jankovic et al. (13) the mean TD score and the mean PIGD score were calculated separately using the UPDRS (mean tremor score: items 16 of Part II and items 20 and 21 of Part III; mean PIGD score: items 13–15 of Part II and items 29 and 30 of Part III), and the ratio between the two (TD/PIGD ratio) was used to classify motor subtypes. In the present study, this method was referenced and modified as follows: a single cutoff of 1.0 for the TD/PIGD ratio was used as the sole boundary (ratio ≤1.0 classified as the PIGD group, and ratio >1.0 classified as the TD group), without a separate indeterminate (mixed) group—that is, patients in the intermediate range (1.0–1.5) of the original classification were incorporated into the TD group. During the same period, volunteers without neurological or psychiatric diseases matched for age and sex were recruited from the community as the HC group. Ultimately, 36 PD patients were included: 19 patients with PIGD subtype, 17 patients with TD subtype, and 19 age-, sex-, and education-matched healthy subjects.

### 2.4 Functional magnetic resonance imaging data acquisition

Data was acquired using a Discovery MR750 3.0 T magnetic resonance scanner manufactured by GE Medical Systems, United States, equipped with a 32-channel head coil. Subjects were instructed to keep their eyes closed during scanning, remain awake and relaxed, and refrain from active thought. Foam pads were used to fix the head and minimize head movement. All patients underwent both 3D-T1 and resting-state BOLD sequence acquisition. Parameters were as follows:3D-T1 images were obtained using a magnetization preparation rapid gradient echo sequence: repetition time = 1900 ms, echo time = 2.13 ms, inversion time = 900 ms, slice thickness = 1 mm, flip angle = 9°, field of view = 256 mm × 256 mm, voxel size = 1 mm × 1 mm × 1 mm, total images = 176. BOLD sequences used echo-planar imaging for T2”weighted imaging: repetition time = 2000 ms, echo time = 30 ms, field of view = 225 mm × 225 mm, flip angle = 90°, matrix = 64 × 64, slice thickness = 3.5 mm, inter-slice gap = 0.7 mm, voxel size = 3.5 mm × 3.5 mm × 4.2 mm, number of slices = 31, total images = 179.

### 2.5 rs-fMRI data preprocessing

Resting-state functional magnetic resonance imaging (rs-fMRI) data were preprocessed using DPABI v4.1, a toolbox based on Statistical Parametric Mapping 12 (SPM12). The preprocessing pipeline was as follows: (1) Format conversion of raw imaging data; (2) The first 10 time points were discarded to minimize the effects of unstable magnetic field at the beginning of scanning and subjects’ adaptation to the scanning environment; (3) Slice-timing correction was performed to compensate for temporal differences in slice acquisition; (4) Realignment for head-motion correction. Subjects with translation >5 mm or rotation >5° in any direction were excluded to mitigate potential confounding effects of head motion; (5) Spatial normalization. The DARTEL algorithm was applied for spatial registration and normalization to warp functional images into standard MNI space; (6) Spatial smoothing using a Gaussian kernel with a full width at half maximum (FWHM) of 6 mm; (7) Removal of linear drift to reduce non-neuronal components such as time-dependent low-frequency drift during scanning; (8) Nuisance covariates including white matter signal, cerebrospinal fluid signal, and Friston-24 head-motion parameters were regressed out by linear regression. Global signal was not regressed in the present study to avoid spurious alterations in inter-regional functional correlations introduced by global signal regression; (9) Band-pass filtering within 0.01–0.08 Hz was carried out to suppress low-frequency drift and high-frequency physiological noise, while preserving the dominant low-frequency fluctuations of resting-state BOLD signals; and (10) Data quality control, including visual inspection of image registration and spatial normalization, assessment of head-motion parameters, and exclusion of subjects who failed the predefined head-motion criteria.

### 2.6 Independent component analysis

Preprocessed rs-fMRI data were decomposed using GIFT v4.0b software.^1^ For ICA decomposition, the BOLD data from all participants (36 PD patients and 19 healthy controls, *N* = 55) were concatenated and jointly decomposed into 30 independent components, ensuring that the resulting components were derived from the same subject space and that the identified networks were comparable across the three groups. ICA decomposes data into spatially independent components (ICs), each with unique spatial maps and temporal profiles. The specific process was as follows: first, the minimum description length (MDL) criterion embedded in GIFT was used to estimate the dimensionality of independent components, and the final number of decomposed independent components was set to 30. MDL is an information-theoretic criterion that balances model goodness-of-fit against model complexity, selecting the number of components that minimizes the total description length of the data, thereby avoiding both over- and under-fitting (14). Next, the Infomax algorithm was used to decompose group-level fMRI data into 30 ICs. The ICASSO algorithm^2^ was employed to improve the stability of ICA decomposition by repeating ICA computation 100 times, and stable ICs were obtained based on clustering outputs. Subsequently, the 30 derived ICs were screened. Visual inspection was performed according to the spatial patterns of each IC. Artifactual ICs predominantly distributed in non-gray-matter regions including ventricles, white matter, blood vessels and brain edges, as well as components contaminated by obvious head-motion or other physiological noise, were excluded. For the remaining ICs, multiple regression analysis was implemented in GIFT between spatial maps of each IC and predefined large-scale functional brain network templates. Spatial regression coefficients were calculated to evaluate spatial matching between each IC and canonical brain networks. Manual identification and classification were performed by comprehensively considering regression coefficients and spatial characteristics of ICs. Multiple ICs with distinct spatial features corresponding to the same canonical functional network were all retained. After screening, 12 valid ICs were finally obtained, which were assigned to nine functional brain networks: Auditory Network (AN), Primary Visual Network (PVN), Higher Visual Network (HVN), Default Mode Network (DMN), Left Frontoparietal Network (LFPN), Right Frontoparietal Network (RFPN), Dorsal Attention Network (DAN), Ventral Attention Network (VAN), and Sensorimotor Network (SMN) (Figure 1). Subsequent dFNC analysis was performed using the time-courses of all 12 screened valid ICs. Time-varying correlations between time-courses of different ICs were computed to characterize dynamic FC patterns among functional network components.

**Figure 1:**
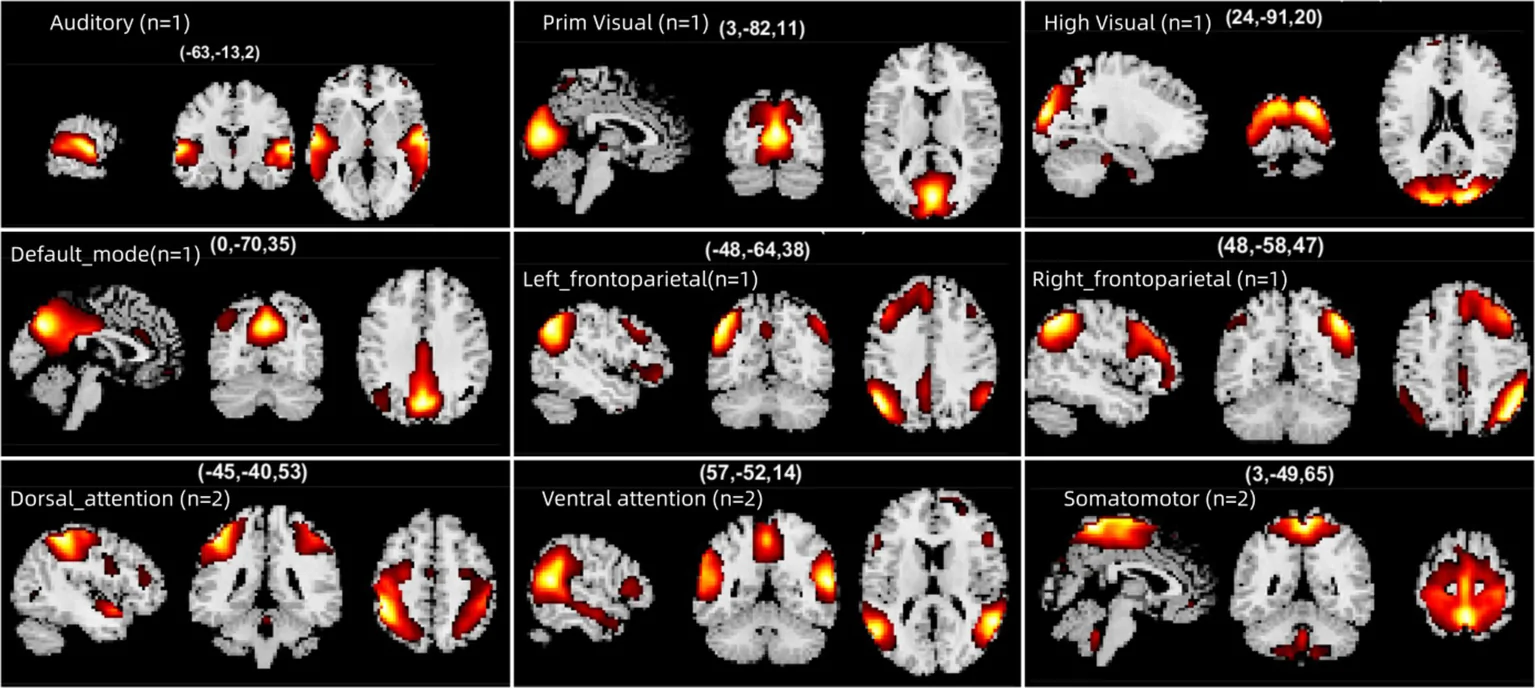
Nine functional networks identified through group-level ICA. Spatial maps of the nine intrinsic functional networks decomposed via group-ICA using the GIFT toolbox. Each panel displays the spatial pattern of one functional network, including the Auditory network, Primary Visual network, High-order Visual network, Default-Mode network, Left Frontoparietal network, Right Frontoparietal network, Dorsal Attention network, Ventral Attention network, and Somatomotor network. The peak MNI coordinates for each independent component are annotated at the top of each sub-panel. Warm-color overlays indicate regions with high component weights. *n* denotes the number of independent components corresponding to each functional network. According to the anatomical and functional characteristics of spatial maps derived from independent components (ICs), a total of 12 ICs were categorized into 9 functional networks: AN, Auditory Network; PVN, Primary Visual Network; HVN, High-Order Visual Network; DMN, Default Mode Network; LFPN, Left Frontoparietal Network; RFPN, Right Frontoparietal Network; DAN, Dorsal Attention Network; VAN, Ventral Attention Network; SMN, Sensorimotor Network.

### 2.7 Dynamic functional connectivity

dFNC for each subject was calculated using the sliding window method and k-means clustering method. A sliding time window of 30 TR was applied to segment resting-state time series data. The previously identified 9 network components were used as regions of interest (ROI), with step size = 1 TR, to calculate dFNC for each subject. The resulting dFNC matrices were clustered using K-means clustering method, with cityblock (Manhattan distance) as the clustering distance metric. According to the elbow criterion, optimal clustering resulted in 4 classes of states, with 1,000 iterations. All matrices for each subject were assigned to one of the 4 specific states.

Time state indicators for each subject were calculated to assess temporal attributes of dFNC states, including: (1) State Frequency (F): ratio of time windows where a specific state occurred to total time windows; (2) Number of Transitions (NT): total number of transitions between different states; (3) Mean Duration Time (MDT): number of time windows a state appeared divided by total occurrences of that state; and (4) Transition Matrix (TM): state transition probability matrix.

### 2.8 Dynamic graph theory

After rs-fMRI data preprocessing, the Automated Anatomical Labeling-116 (AAL-116) atlas was used for whole-brain parcellation, yielding a total of 116 regions of interest (ROIs). The mean BOLD time-series across all voxels within each ROI were extracted as the representative signal for that brain region. The sliding-window approach was then applied to construct dynamic FC networks, with a window length of 30 TR and a step size of 1 TR. Within each sliding window, pairwise Pearson correlation analyses were performed on the mean BOLD time-series of the 116 ROIs to generate a symmetric 116 × 116 FC matrix. For subsequent graph-theoretical analyses, only positive correlations were retained. Negative connections with Pearson correlation coefficients below zero were set to zero. GRETNA (Graph Theoretical Network Analysis Toolbox) was utilized to construct undirected binary networks based on positive correlations. To mitigate the influence of arbitrary single-threshold selection, the network sparsity range was set from 0.05 to 0.40 with an interval of 0.01. The FC matrix was binarized at each sparsity threshold: retained connections were assigned a value of 1 and remaining connections were set to 0. Global and nodal graph-theoretical metrics were computed correspondingly under each sparsity level. Furthermore, GRETNA was used to calculate the area under the curve (AUC) for each graph-theoretical metric across the sparsity range of 0.05–0.40, producing integrated topological metrics relatively independent of specific threshold choices. For each subject, graph-theoretical metrics were obtained for every sliding window. The temporal variability of each metric across windows was further calculated to characterize dynamic fluctuations in brain network topological properties. These derived metrics were submitted to subsequent statistical analyses. The k-means clustering algorithm was implemented using the cityblock (L1) distance metric rather than Euclidean distance. The cityblock metric calculates the absolute difference between feature vectors and is less sensitive to outlier values, rendering it suitable for high-dimensional neuroimaging data (9, 15). To determine the optimal cluster number *k*, we adopted the elbow criterion based on the ratio of dispersion (within-group / between-group), serving as our cluster validity index. This index quantifies the balance between within-cluster compactness and between-cluster separation. The elbow criterion identifies the optimal *k* by locating the point where the rate of reduction in the dispersion ratio slows markedly, representing a trade-off between model complexity and clustering quality. We evaluated k values ranging from 2 to 10. The corresponding elbow plot illustrating the ratio of dispersion across tested k values was provided as Supplementary Figure S1, which identified k = 4 as the optimal cluster number.

### 2.9 Statistical analysis

Clinical data were analyzed using SPSS version 31.0 software.^3^ Continuous data approximately following normal distribution were expressed as mean ± standard deviation (*x̄*± *s*), with two-sample *t*-test used for comparison between 2 groups and one-way analysis of variance (ANOVA) for comparisons among 3 groups or more, followed by LSD-*t* test for further pairwise comparisons. Non-normally distributed continuous data were expressed as median [first quartile, third quartile] ([*M*(*Q1*, *Q3*)]), with Mann–Whitney *U* test for 2-group comparisons and Kruskal-Wallis *H* test for multi-group comparisons, followed by Bonferroni correction for pairwise comparisons. Categorical data were expressed as number (%). The Chi-square (*χ*^2^) test used for inter-group comparisons. *p* < 0.05 was considered statistically significant. Group analysis of dFNC was performed using the Stats module in GIFT software package. Inter-group comparisons of temporal attributes and connection strength under different states were performed using the general linear model (GLM), with age, sex, years of education included as covariates. Multiple comparison correction was performed using the false discovery rate (FDR). Dynamic graph theory analysis was based on 116 ROIs delineated by the AAL116 brain region template. Pearson correlation coefficients between 116 ROI time series were calculated pairwise within each time window to construct a 116 × 116 FC matrix. Brain network graph theory attributes (including global metrics and node metrics) were calculated using GRETNA based on this matrix. To reduce the impact of network density selection on results, network construction was performed using sparsity threshold ranges from 0.05–0.40 (step size 0.01), and area under the curve (AUC) of graph theory indicators within this threshold range was calculated. Variability of graph theory indicators across all time windows for each subject was further calculated.

GRETNA was used to calculate PD-brain network graph theory attributes, including global metrics and node metrics. Age, sex, and years of education were used as covariates, and partial correlation analysis was conducted to examine correlations between dFNC attributes (functional connection strength, time state indicators) of PIGD group patients and clinical scales. *p* < 0.05 was considered statistically significant.

## 3 Results

### 3.1 Comparison of clinical characteristics

A total of 55 participants were enrolled in this study, including 19 patients in the PIGD group, 17 patients in the TD group, and 19 individuals in the HC group. There were no significant differences in age, gender or education level among the three groups (*p* > 0.05). Statistically significant differences were observed in scores of the MMSE and MoCA (*p* < 0.05). All resting-state functional magnetic resonance imaging (rs-fMRI) scans were acquired under a unified off-medication state. Specifically, to minimize the potential confounding effect of dopaminergic medication (e.g., levodopa) on resting-state neural activity, all patients were asked to withhold antiparkinsonian medication for approximately 12 h (i.e., overnight withdrawal) before the fMRI session, and the time interval between the last dose and the scan was kept fixed at about 12 h across all participants. This design is in line with the widely used definition of the “practical OFF state” in PD neuroimaging studies, which commonly corresponds to dopaminergic withdrawal of ≥12 h (overnight). In addition, the levodopa equivalent daily dose (LEDD) of each patient was recorded to characterize the daily dopaminergic medication load.

No significant difference in disease duration was found between the PIGD and TD groups. However, significant intergroup differences were detected in the Hoehn-Yahr stage and motor subscore of the UPDRS-III (all *p* < 0.05), with higher scores in the PIGD group than in the TD group. Post-hoc pairwise comparisons of cognitive scales revealed that the MMSE scores decreased sequentially from the HC group to the TD group and then to the PIGD group, and the PIGD group had markedly lower MMSE scores than the HC group, with all differences reaching statistical significance. For MoCA scores, significant differences were only found between the two PD subgroups and the HC group, whereas no significant difference existed between the PIGD and TD groups. Detailed data are presented in Table 1.

**Table 1:** Demographic and clinical characteristics of PIGD group, TD group and HC group.

Item	PIGD (*n* = 19)	TD (*n* = 17)	HC (*n* = 19)	*F*/*χ*^2^/*H/Z*/*t*	*P*
Age (years, x̄±s)	67.32 ± 5.93	67.12 ± 8.03	63.16 ± 3.42	2.840	0.067
Gender (male/female, n)	7/12	7/10	13/6	4.407	0.110
Education duration [years, M(*Q_1_*, Q*_3_*)]	9.0 (5.0, 10.0)	10.0 (8.0, 12.0)	9.0 (6.0, 10.0)	3.712	0.156
Disease duration [months, *M*(*Q_1_*, Q*_3_*)]	48.0 (12.0, 84.0)	48.0 (12.0, 72.0)	–	−1.328	0.184
Hoehn-Yahr stage [*M*(*Q_1_*, Q*_3_*)]	2.0 (2.0, 3.0)	1.5 (1.0, 2.5)	–	−2.751	0.006
UPDRS-III	31.84 ± 17.03	22.59 ± 10.53	–	−2.236	0.025
MMSE	20.0 (14.0, 28.5)	25.0 (21.5, 27.5)	27.0 (27.0, 28.0)	11.773	0.003
MoCA	18.00 (11.0, 26.0)	22.0 (17.0, 24.5)	26.0 (25.0, 26.0)	24.994	<0.001
Patients receiving levodopa, *n* (%)	14 (73.7%)	12 (70.6%)	–	0.046	0.831
Patients receiving dopamine agonists, *n* (%)	11 (57.9%)	9 (52.9%)	–	0.090	0.764
Patients receiving MAO-B inhibitors, *n* (%)	4 (21.1%)	3 (17.6%)	–	0.062	0.801
LEDD (mg/d, x̄±s)	545.17 ± 276.47	296.03 ± 172.09	–	3.20	0.003

MMSE, Mini-Mental State Examination; UPDRS-III, Part III of the Unified PD Rating Scale; LEDD, Equivalent daily dose of levodopa.

### 3.2 Between-group component difference analysis within brain networks

ANOVA was performed based on the GLM using SPM12 software to compare the differences in spatial distribution of component maps across the 9 brain networks among the HC group, PIGD subtype group, and TD subtype group, with age and sex included as covariates to eliminate their confounding effects. The statistical thresholds were set at voxel level *p* < 0.01 and cluster level *p* < 0.05, corrected by Gaussian Random Field (GRF) theory. Post-hoc comparisons among the three groups were conducted using Tukey–Kramer test (Figure 2), as detailed in Table 2. All pairwise contrasts were pre-specified: HC vs. PIGD, HC vs. TD, and TD vs. PIGD. Only HC vs. PD-subtype contrasts reached statistical significance for the right middle temporal gyrus and right angular gyrus components; TD versus PIGD was not statistically significant for the right angular gyrus.

**Figure 2:**
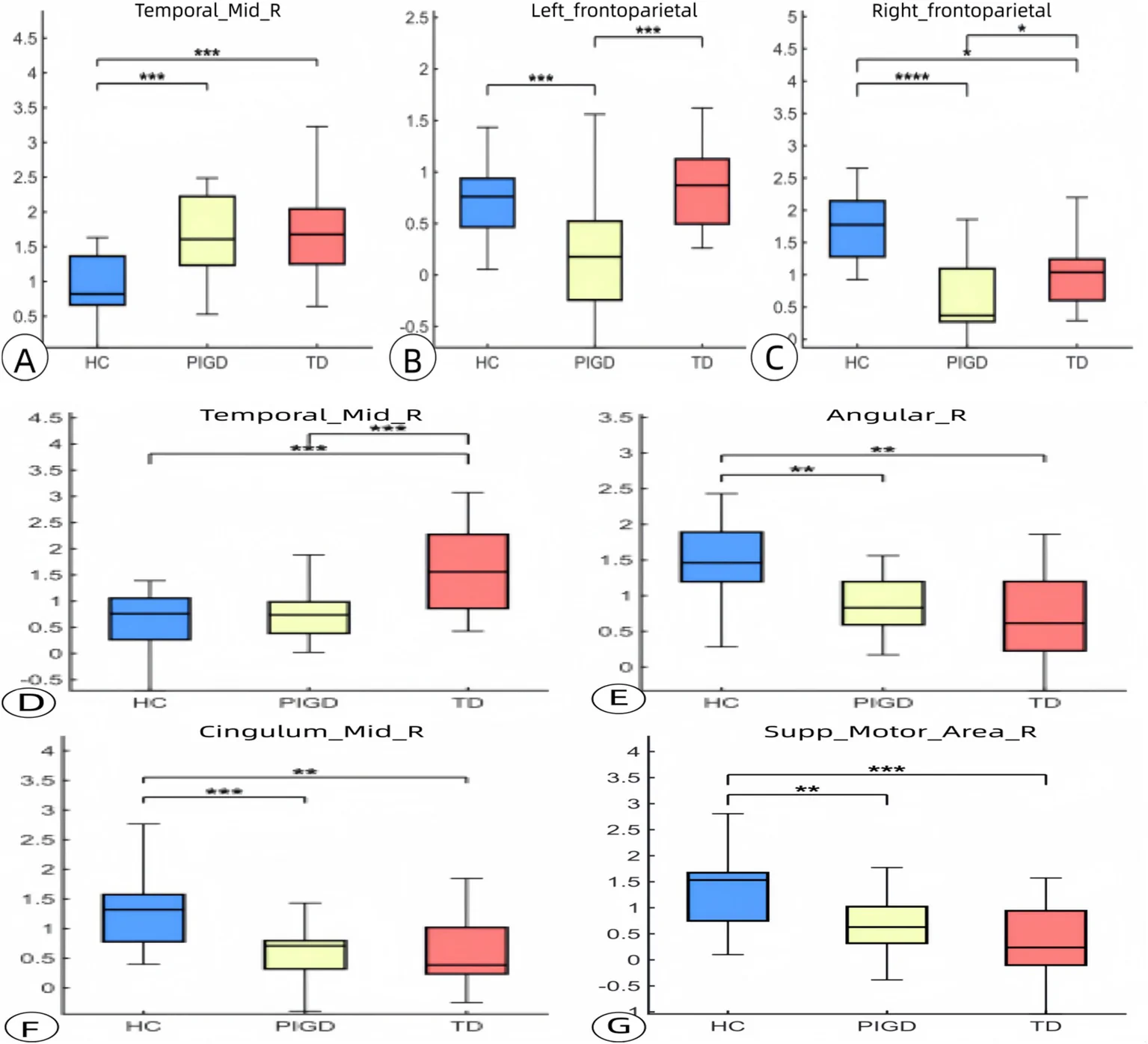
Inter-group comparison of functional component values of brain networks in each group. Box-and-whisker plots illustrate the distribution of functional component values for brain-network-derived metrics among three groups: HC, blue; PIGD, light-yellow, and TD, red. The horizontal line inside each box represents the median; box limits indicate the 25th and 75th percentiles; whiskers extend to the minimum and maximum observed values. **(A–C)** Group comparisons for whole-network component metrics. **(D–G)** Regional component-value comparisons for selected brain regions. **(A)** Auditory-Temporal_Mid_R; **(B)** Left_frontoparietal-Frontal_Sup_L; **(C)** Right_frontoparietal-Frontal_Mid_R; **(D)** Ventral attention-Temporal_Mid_R; **(E)** Ventral attention-Angular_R; **(F)** Somatomotor-Cingulum_Mid_R; **(G)** Somatomotor-Supp_Motor_Area_R. *: *p* < 0.05;**: *p* < 0.01;***: *p* < 0.005.

**Table 2:** Information of brain regions with significant differences in functional network components among groups.

Functional network	Brain region	BA	MNI peak coordinates			Cluster size	*F*
			X	Y	Z		
Auditory network	Right Middle Temporal Gyrus	21/22	60	−39	6	30	8.396
Left frontoparietal network	Left Superior Frontal Gyrus	9/10	−27	33	33	22	8.284
Right frontoparietal network	Right Middle Frontal Gyrus	46/10	27	45	33	44	12.405
Ventral attention network	Right Middle Temporal Gyrus	21/22	48	−45	6	26	9.676
	Right Angular Gyrus	39/40	48	−54	36	25	11.391
Sensorimotor network	Right Middle Cingulate Gyrus	24/32	9	−36	45	31	12.259
	Right Supplementary Motor Area	6	9	12	63	40	8.461

### 3.3 Functional connectivity differences between brain networks

One-way analysis of variance was performed on the FC matrices among the 9 brain networks, with FDR correction applied to the results (*p* < 0.05). The ANOVA results revealed a significant between-group difference between the AN and SMN (*F* = 7.548, FDR-corrected *p* = 0.00137) (Figure 3A). Post-hoc tests demonstrated that FC was strongest in the HC group, followed by the TD group, and weakest in the PIGD group (Figure 3B).

**Figure 3:**
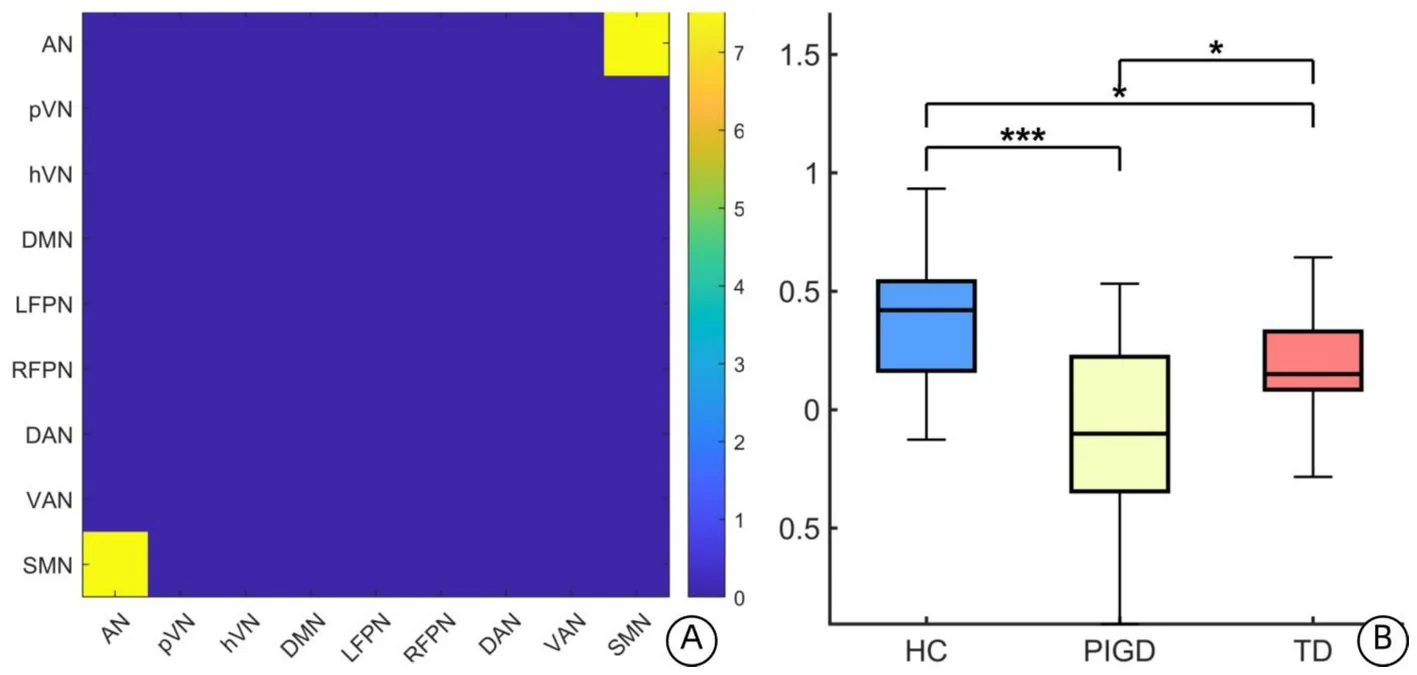
Analysis of functional connectivity differences among 9 brain networks. **(A)** Heatmap illustrating group-level functional connectivity matrix among the nine intrinsic brain networks (AN, PVN, HVN, DMN, LFPN, RFPN, DAN, VAN, SMN). The color bar represents the magnitude of functional-connectivity values, ranging from 0 (dark blue) to 7 (bright yellow). **(B)** Box-and-whisker plot showing between-group comparisons of inter-network functional connectivity values across healthy controls (HC, blue), PIGD subtype (light-yellow), and TD subtype (red). Each box denotes the 25th-75th percentiles; the horizontal line inside each box is the median; whiskers extend to observed minimum and maximum values. Horizontal bars indicate post-hoc pairwise comparisons. *: *p* < 0.05; **: *p* < 0.01;***: *p* < 0.005.

### 3.4 Clustering analysis and dFNC state results

K-means clustering was employed, and four recurring functional connectivity (FC) states were ultimately identified. Across all sliding windows of the full cohort, the overall occurrence frequencies ranked from highest to lowest were: State 2 (38%), State 3 (23%), State 4 (21%), and State 1 (18%). Among the four states, both State 2 and State 3 exhibited a low-connectivity network pattern, with particularly pronounced reductions in FC between the SMN and the DMN, LFPN, and RFPN. In State 1, the DMN demonstrated high-intensity positive FC with bilateral frontoparietal networks (LFPN/RFPN) and the VAN. State 4 showed synchronized increases in FC among the VAN, SMN, and PVN, with enhanced network collaborative activation (Figure 4).

**Figure 4:**
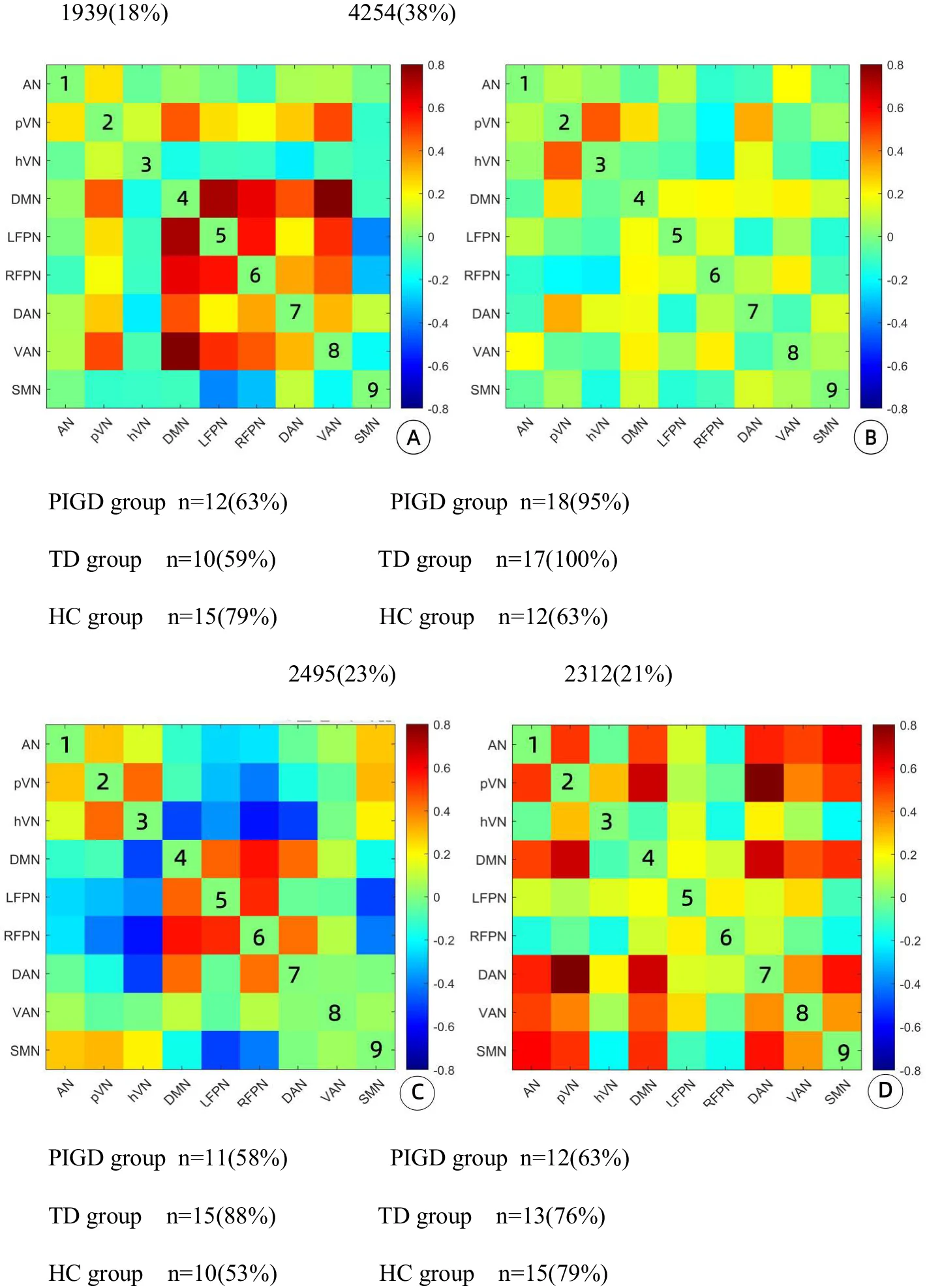
Cluster center matrix for the 4 states in k-means clustering. Cluster-centroid functional-connectivity matrices for the four recurring dynamic functional network connectivity (dFNC) states. Rows and columns correspond to the nine brain networks. Color bars represent functional-connectivity coefficient values (range −0.8 to 0.8; red for positive correlations, blue for negative correlations). **(A)** State 1; **(B)** State 2; **(C)** State 3; **(D)** State 4. Text below each matrix reports the proportion of participants exhibiting each state within HC, PIGD and TD groups; absolute subject numbers are also provided.

### 3.5 Comparison of dFNC state patterns across brain networks

Comparison of temporal attributes of dFNC states among the three groups showed that state occurrence frequency differed significantly only in State 4 among the three groups (*F* = 4.855, FDR-corrected *p* = 0.047). Post-hoc tests indicated that the occurrence frequency of State 4 was significantly higher in the HC group compared to the PIGD group, and exhibited a trend toward being higher than that in the TD group, whereas no significant difference was observed between the two PD subtypes. State transition count: No significant difference in state transition counts was found across the three groups (*F* = 0.515, *p* = 0.600), suggesting no significant difference in overall dynamicity of brain network state transitions among the three groups. Mean dwell time: Differences in mean dwell time for State 4 were observed among the three groups at uncorrected level only (*F* = 3.376, *p* = 0.042), but did not reach statistical significance after FDR correction. Post-hoc tests revealed an overall trend with the HC group showing higher values than both PD subtypes, but no significant differences between any pair of groups. State transition probability matrix: Transition probabilities among the four states showed no significant differences among the three groups (non-significant before and after correction) (Figure 5).

**Figure 5:**
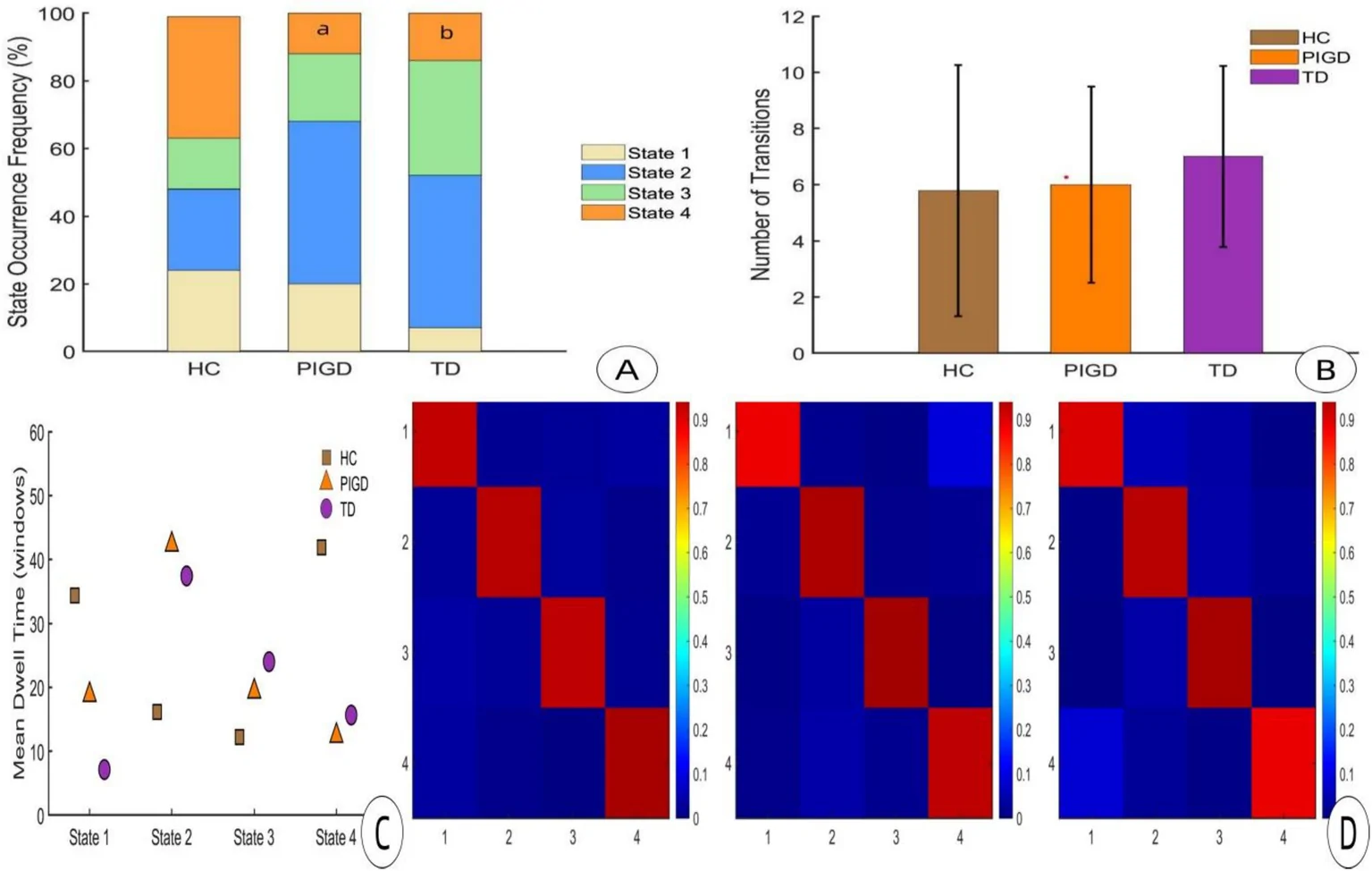
Comparison results of temporal attributes and intensity of functional connectivity status among the PIGD group, the TD group and the HC group **(A)** Stacked bar plot showing state occurrence frequency (%) for each dFNC state across the three groups. Different colors represent State 1–4; letters a, b denote significant between-group differences. **(B)** Bar plot comparing the mean number of state transitions among groups; error bars represent standard deviation. **(C)** Scatter plot illustrating mean dwell time (in time-windows) for each dFNC state across HC, PIGD and TD groups. Symbols: squares = HC, triangles = PIGD, circles = TD. **(D)** Group-specific state transition probability matrices for HC, PIGD, TD, respectively. Color bar denotes transition probability values (0–0.9; red = higher transition probability, blue = lower transition probability). Note. Compared with the PIGD group, ^a^ *p* < 0.05; compared with the TD group, ^b^ *p* < 0.05.

### 3.6 Analysis of topological properties of brain functional networks

#### 3.6.1 Global metrics analysis

ANOVA was performed on functional connections across all brain nodes. At a significance threshold corrected by FDR (*p* < 0.05), no brain regions showed significant between-group differences. The comparisons of global graph-theoretical network attributes for the three groups are detailed in Table 3.

**Table 3:** Comparison of global attributes of the three groups.

Global properties	PIGD	TD	HC	*F*	*P* (FDR)
Eg	0.000066 ± 0.000054	0.000069 ± 0.000054	0.000066 ± 0.000042	0.0304	0.970
Eloc	0.000059 ± 0.000031	0.000052 ± 0.000036	0.000048 ± 0.000026	0.3968	0.675
Cp	0.000043 ± 0.000027	0.000047 ± 0.000028	0.000046 ± 0.000018	0.1870	0.830
Lp	0.002578 ± 0.002283	0.002974 ± 0.002763	0.002606 ± 0.001559	0.2415	0.786
λ	0.000130 ± 0.000121	0.000146 ± 0.000116	0.000114 ± 0.000074	0.4294	0.653
γ	0.012513 ± 0.006174	0.011831 ± 0.003280	0.011496 ± 0.003830	0.2171	0.806
σ	0.010287 ± 0.004936	0.009920 ± 0.002887	0.009752 ± 0.003298	0.0927	0.912

Eg, global efficiency; Eloc, local efficiency; Cp, clustering coefficient; Lp, characteristic path length.λ, normalized characteristic path length; γ, normalized clustering coefficient; σ, small-worldness; In this study, the global topological properties refer to the AUC of the metrics calculated across different sparsity thresholds (0.05–0.40). The AUC was used to integrate results from multiple thresholds and avoid bias caused by selecting a single threshold.

#### 3.6.2 Node metric analysis

One-way ANOVA was performed on the graph theoretical properties of node maps across the three groups. No significant differences were found after FDR correction; however, at the uncorrected *p* < 0.05 threshold, differences among the three groups exhibited the following regional distribution patterns: Cuneus_R demonstrated differential trends in both Betweenness Centrality (BC) and Node Efficiency (NE), while Precuneus_R showed differential trends in both Degree Centrality (DC) and NE, as detailed in Tables 4, 5 and Figure 6. Further Tukey–Kramer post-hoc tests revealed that the NE of Cuneus_R in the PIGD group was significantly higher than that in the HC group (uncorrected *p* < 0.05); additionally, the DC of Precentral_R in both the PIGD group and TD group were significantly higher than in the HC group (respectively uncorrected *p* < 0.05 and uncorrected *p* < 0.01). The brain regions exhibiting differential trends in BC were primarily distributed in the frontal lobe, occipital lobe, temporal lobe, and cerebellum; the regions showing differential trends in DC were concentrated in the frontal lobe, parietal lobe, temporal lobe, and cerebellum, particularly involving multiple subregions of the cerebellar hemispheres and vermis; NE differential trend regions were mainly located in the occipital lobe and parietal lobe, with partial involvement of temporal lobe regions (Figure 7).

**Table 4:** Summary of intergroup differences in nodal topological properties.

Node properties	ROI number	Brain region	Functional network	*F*	*P*
NE	46	Right Cuneus	PVN	4.109	0.022
	50	Right Superior Occipital	HVN	5.851	0.005
	61	Left Inferior Parietal Lobule	DAN	4.551	0.015
	62	Right Inferior Parietal Lobule	DAN	3.766	0.030
	68	Right Precuneus	DAN	4.742	0.013
	82	Right Superior Temporal Gyrus	AN	3.345	0.043
BC	3	Left Superior Frontal Gyrus	LFPN	6.542	0.003
	21	Left Olfactory Cortex	DMN	3.274	0.046
	26	Right Medial Orbital Frontal Gyrus	DMN	7.247	0.002
	43	Left Calcarine Cortex	PVN	3.980	0.025
	46	Right Cuneus	PVN	4.136	0.022
	55	Left Fusiform Gyrus	HVN	3.227	0.048
	57	Left Postcentral Gyrus	SMN	4.087	0.023
	105	Left Cerebellar Lobule 9	CN	3.384	0.042
DC	2	Right Precentral Gyrus	SMN	3.203	0.049
	6	Right Superior Orbital Frontal Gyrus	DMN	7.156	0.002
	22	Right Olfactory Cortex	DMN	6.414	0.003
	40	Right Parahippocampal Gyrus	DMN	3.399	0.041
	58	Right Postcentral Gyrus	SMN	3.424	0.040
	67	Left Precuneus	DAN	3.536	0.037
	68	Right Precuneus	DAN	7.172	0.002
	93	Left Cerebellar Crus 2	CN	3.773	0.030
	106	Right Cerebellar Lobule 9	CN	3.855	0.028
	110	Cerebellar Vermis Lobule 3	CN	6.957	0.002
	113	Cerebellar Vermis Lobule 7	CN	4.646	0.014
	115	Cerebellar Vermis Lobule 9	CN	4.112	0.022

PVN, Primary Visual Network; HVN, High-order Visual Network; DAN, Dorsal Attention Network; AN, Auditory Network; LFPN, Left Fronto-Parietal Network; SMN, Sensorimotor Network; DMN, Default Mode Network; CN, Cerebellar Network.

**Table 5:** Comparison of node attributes of the three groups.

Brain region	*F*					*P*				
	Bc	Dc	NCp	NLe	Ne	Bc	Dc	NCp	NLe	Ne
Right cuneus	4.136	1.959	2.636	3.228	4.109	0.022	0.152	0.082	0.022	0.048
Right precuneus	0.016	7.172	1.807	4.966	4.741	0.984	0.002	0.175	0.013	0.011

BC, Betweenness Centrality; DC, Degree Centrality; NCp, Node Clustering Coefficient; NLe, Node Local Efficiency; Ne, Node Efficiency.

**Figure 6:**
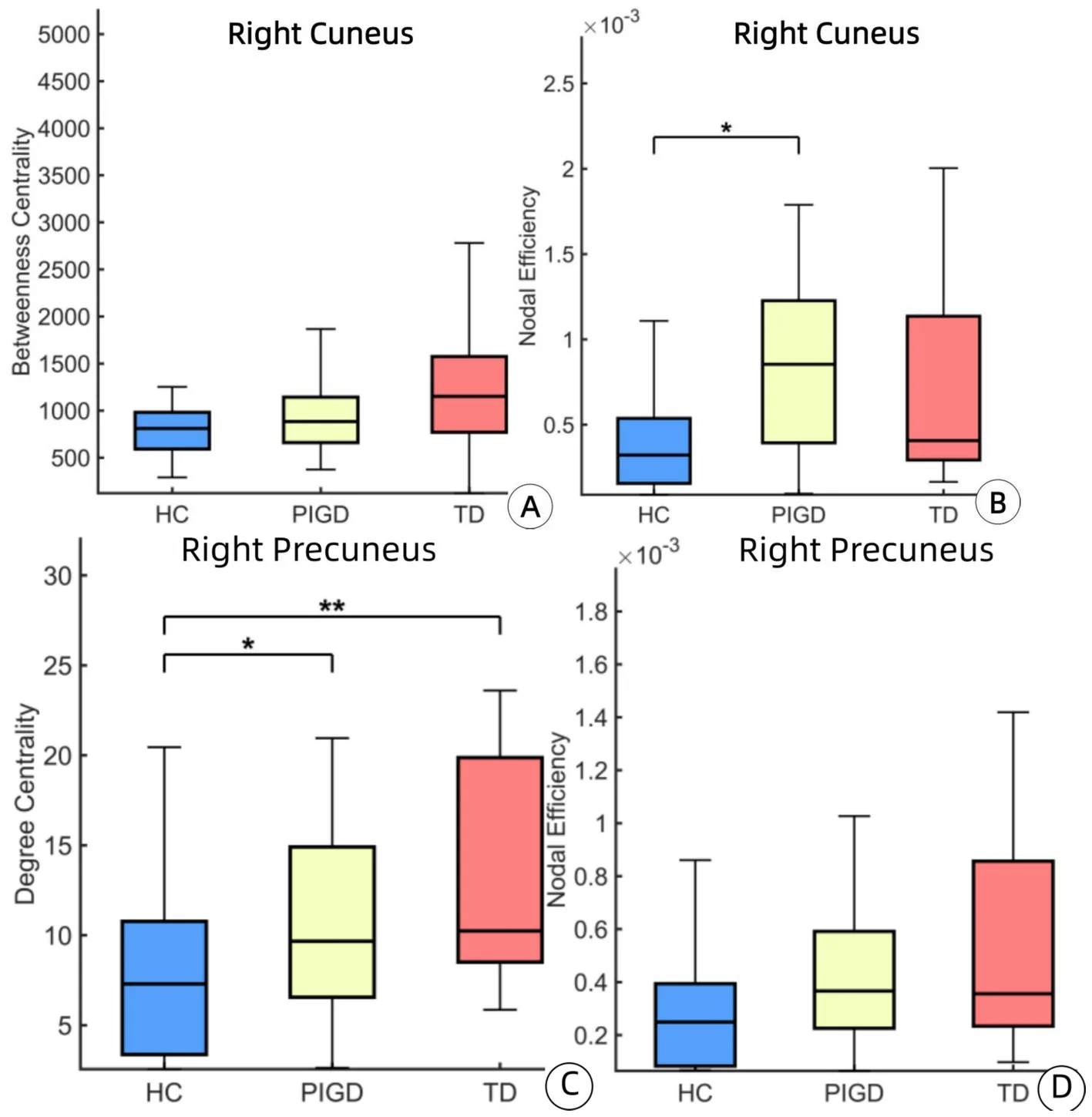
Comparison of nodal topological properties among the three groups. Box-and-whisker plots illustrate group differences in nodal graph-theory metrics. **(A)** Betweenness centrality of the right cuneus; **(B)** Nodal efficiency of the right cuneus; **(C)** Degree centrality of the Right Precuneus; **(D)** Nodal efficiency of the Right Precuneus. Boxes represent the interquartile range (25th-75th percentiles); the horizontal line inside each box indicates the median. Whiskers extend to the minimum and maximum observed values. Blue boxes: HC; light-yellow boxes: PIGD subtype patients; red boxes: TD subtype patients. Horizontal brackets denote post-hoc pairwise comparisons. *: *p* < 0.05;**: *p* < 0.01;***: *p* < 0.005.

**Figure 7:**
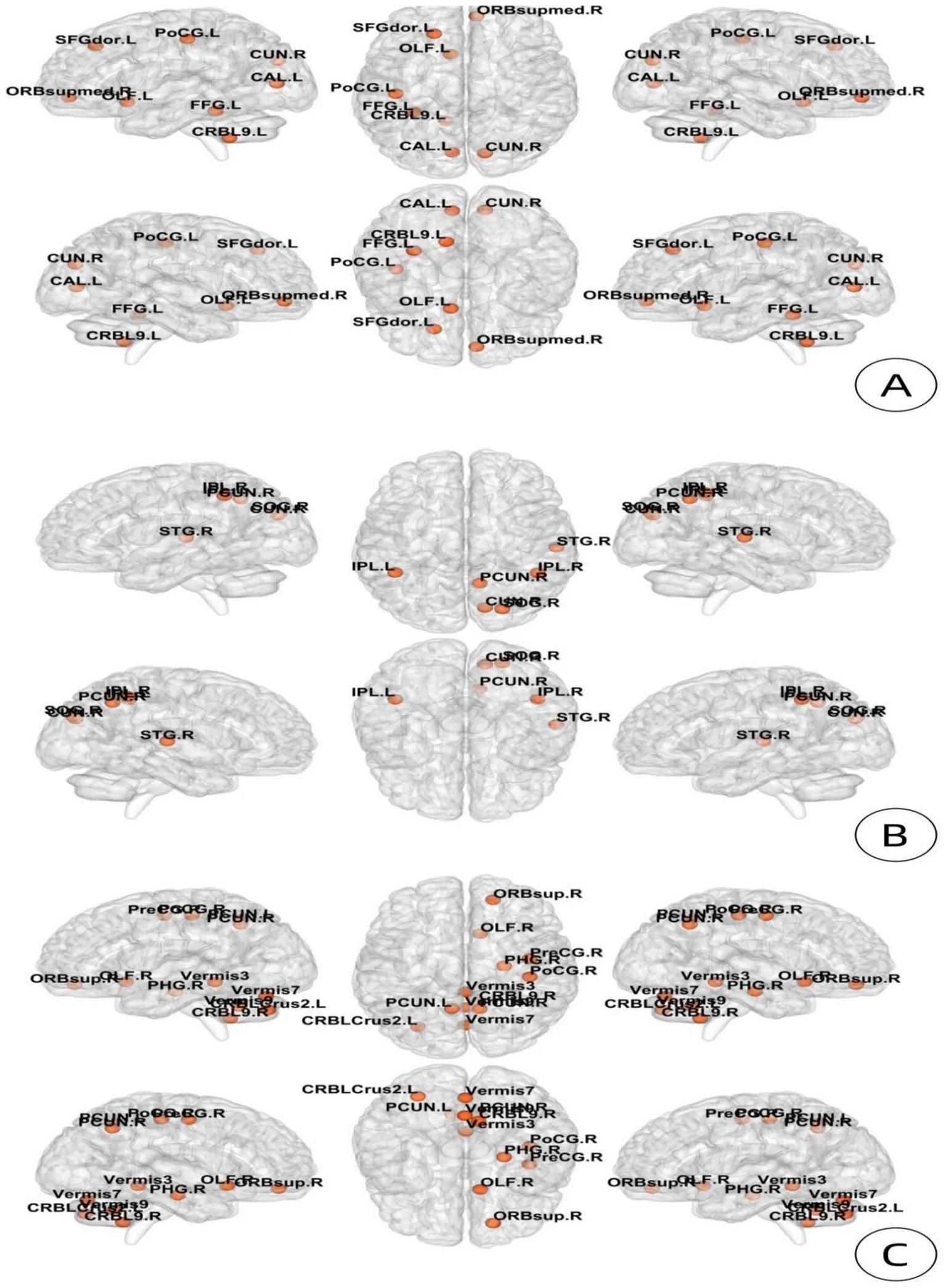
Distribution of brain regions with differences in nodal graph theory properties among three groups. Brain surface renderings display brain nodes with statistically significant differences in nodal topological metrics among HC, PIGD, and TD groups. Orange dots mark the location of discrepant brain regions. **(A–C)** Different sets of brain nodes corresponding to distinct nodal metrics. **(A)** Betweenness Centrality. **(B)** Nodal efficiency. **(C)** Degree CentralityCun_R: Cuneus_R; Occ_Sup_R: Occipital_Sup_R; Par_Inf_L: Parietal_Inf_L; Par_Inf_R: Parietal_Inf_R; PreCun_R: Precuneus_R; Temp_Sup_R: Temporal_Sup_R; Front_Sup_L: Frontal_Sup_L; Olf_L: Olfactory_L; Front_Med_Orb_R: Frontal_Med_Orb_R; Cal_L: Calcarine_L; Fus_L: Fusiform_L; PostC_L: Postcentral_L; Cereb_9_L: Cerebelum_9_L; PreC_R: Precentral_R: Front_Sup_Orb_R: Frontal_Sup_Orb_ROlf_R: Olfactory_R; ParaHipp_R: ParaHippocampal_R; PostC_R: Postcentral_R; PreCun_L: Precuneus_L; Cereb_Crus2_L: Cerebelum_Crus2_L; Cereb_9_R: Cerebelum_9_R; Verm_3: Vermis_3;Verm_7: Vermis_7; Verm_9: Vermis_9.

### 3.7 Correlation analysis between dFNC metrics and clinical characteristics

After adjusting for the effects of age, sex, and years of education, the fractional occupancy of State 2 in the TD group showed a positive correlation with Hoehn-Yahr staging scores (*r* = 0.506, *p* = 0.038). In the PIGD group, the mean dwell time in State 3 demonstrated a negative correlation with UPDRS-III motor scores (*r* = −0.465, *p* = 0.045). No significant correlations were observed between other temporal property metrics, FC strength metrics, and clinical characteristic scores (*p* > 0.05) (Figure 8).

**Figure 8:**
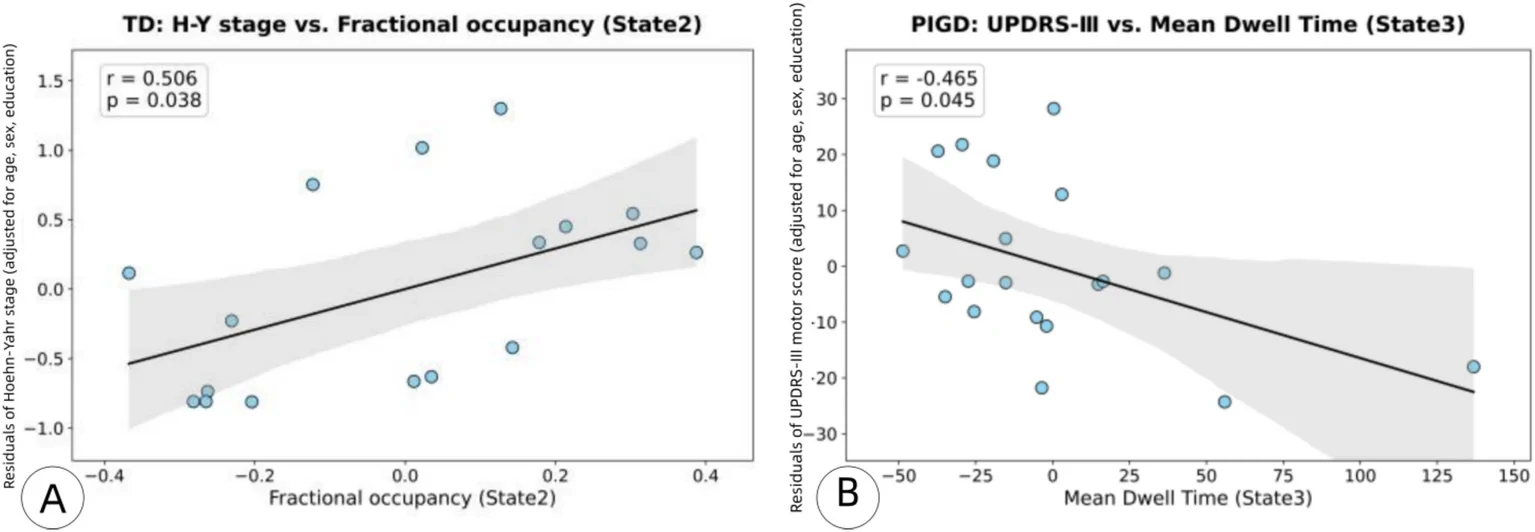
Correlation analyses between dynamic functional connectivity indices and clinical characteristics in the TD and PIGD Subgroups. Scatter plots with fitted regression lines and shaded 95% confidence intervals illustrate significant partial correlation results. Y-axes represent residuals after adjustment for age, sex, and years of education (not raw clinical scores). **(A)** Correlation between Hoehn-Yahr (H-Y) stage residuals and fractional occupancy of State 2 in the TD subgroup. Partial correlation coefficient (*r* = 0.506, *p* = 0.038). **(B)** Correlation between UPDRS-III motor score residuals and mean dwell time of State 3 in the PIGD subgroup. Partial correlation coefficient (*r* = −0.465, *p* = 0.045). **(A)** Correlation analysis between the number of state transitions in State 2 and Hoehn-Yahr stage scores in the TD group; **(B)** Correlation analysis between the MDT in State 3 and UPDRS-III scores in the PIGD group.

## 4 Discussion

### 4.1 Differences between PD group and HC group

In the present study, ICA combined with dFNC and dynamic graph theory was adopted to compare the dynamic characteristics of brain functional networks among the PIGD subtype, TD subtype and HC group from a dynamic perspective. The results revealed that compared with the HC group, the overall PD group (PIGD and TD subgroups) exhibited significant abnormalities in the spatial distribution of five core brain networks, namely the AN, LFPN, RFPN, VAN and SMN. Specifically, component values of the right middle temporal gyrus within the AN and VAN, as well as the right middle cingulate gyrus and right supplementary motor area within the SMN, were markedly higher in PD subtypes (TD and PIGD) than those in the HC group. These findings may reflect compensatory overactivation of brain networks subserving auditory information processing, attentional function and sensorimotor regulation in PD patients, consistent with core clinical manifestations of PD including hypophonia, slowed auditory response, bradykinesia and muscular rigidity. The results were also in line with previous studies (16, 17), confirming that abnormal spatial distribution of brain networks serves as a crucial neuroimaging basis for functional deficits in PD. In terms of FC, the strength of FC between AN and SMN was significantly reduced in the PD group relative to the HC group, suggesting impaired coordination of auditory-motor integration. This may account for speech-motor disorders and desynchronization between movements and auditory signals in PD patients. dFNC analysis demonstrated that the overall PD group showed a notably lower occurrence rate of State 4 (characterized by enhanced synergistic activation of VAN, SMN and PVN), while States 2 and 3 (low-connectivity network patterns) dominated the functional states. These observations implied a decline in the synergistic activation capacity of brain networks in PD patients, with low synergistic activation patterns becoming the predominant brain functional state, which may underlie the impaired coordination of cognitive and motor functions in PD. Multiple prior studies have verified that PD patients spend substantially more time in sparse/low-connectivity states (State 2 and 3) and exhibit lower occurrence rates of highly connected states (State 1 and 4) compared with healthy individuals (18, 19). For nodal graph theoretical metrics, no significant intergroup differences were observed across the three groups after FDR correction. However, trends of differences in topological properties of brain regions were detected at the uncorrected statistical level. The nodal efficiency (NE) of the right cuneus (Cuneus_R) was significantly higher in the PIGD group than in the HC group. Meanwhile, the degree centrality (DC) of the Right Precuneus was elevated in both PIGD and TD groups relative to the HC group. These results suggested abnormal activation of the motor cortex in PD patients, which may be associated with motor dysfunction (20). In summary, the overall differences in brain function between the HC group and PD group were characterized by abnormal spatial distribution of multiple brain networks, weakened FC of specific networks, declined synergistic activation capacity of dFNC, and elevated activation of the motor cortex. Collectively, these brain functional abnormalities constitute the neuroimaging substrate for cognitive and motor impairments in PD, providing objective evidence for the general diagnosis of this disease.

### 4.2 Differences between two motor subtypes of PD and their correlations with cognitive and motor scale scores

PD presents prominent clinical heterogeneity and is clinically classified into two motor subtypes based on predominant motor symptoms: TD and PIGD. Previous studies have demonstrated that the PIGD subtype is mainly manifested as postural and gait disturbances as well as bradykinesia, whereas the TD subtype is dominated by resting tremor. With regard to cognitive function, the PIGD subtype suffers from more severe executive dysfunction, while cognitive impairment is relatively mild in the TD subtype (21). Using rs-fMRI (rs-fMRI), the present study identified subtype-specific alterations in brain network features, which were strongly correlated with clinical scale scores. These findings uncovered the distinct neuropathological mechanisms underlying varied clinical phenotypes of the two subtypes.

#### 4.2.1 Subtype-specific differences in spatial distribution of brain networks

Our results showed that the component value of the left superior frontal gyrus within LFPN was significantly lower in the PIGD group than in the TD group, which was consistent with previous reports. As a core network for executive control, LFPN is involved in higher-order cognitive functions such as working memory, inhibitory control and task switching. Accumulated evidence has indicated that LFPN dysfunction correlates with the severity of cognitive impairment, particularly executive dysfunction, in PD patients (22). In this study, the PIGD group obtained lower MMSE scores than the TD group, indicating more severe cognitive deficits, which corroborated the attenuated LFPN function in the PIGD subgroup. Nevertheless, no significant between-group difference was found in MoCA scores. This phenomenon may be explained by two factors. First, the limited sample size (19 PIGD patients and 17 TD patients) may have insufficient statistical power to detect subtle cognitive differences between the two subtypes. Second, although MoCA is sensitive to executive dysfunction, it may fail to capture mild cognitive changes induced by specific LFPN lesions. Further studies combining specialized executive function assessments such as the Frontal Assessment Battery (FAB) are required to validate the quantitative association between frontoparietal network function and cognitive impairment. The component value of the right middle frontal gyrus in RFPN exhibited a gradual decreasing trend across groups: HC group > TD group > PIGD group. This gradient pattern was highly consistent with higher Hoehn-Yahr staging and Unified PD Rating Scale Part III (UPDRS-III) motor scores in the PIGD group. RFPN participates in motor planning, sequence programming and movement execution, and its dysfunction directly leads to postural gait disorders and bradykinesia. Systematic neuroanatomical reviews have documented subtype-specific alterations in white matter microstructure in PIGD patients, including reduced nerve fiber density in the corona radiata and multiple white matter tracts, which are more extensive compared with the TD subtype (23, 24). Given that postural instability and gait difficulty are the hallmark symptoms of the PIGD subtype, the markedly reduced RFPN function in this subgroup suggests that RFPN may serve as a relevant neuroimaging indicator for differentiating two PD subtypes. This finding also provides a neurophysiological explanation for the clinical features of PIGD, including faster disease progression and higher fall risk. The component value of the right angular gyrus within the VAN showed a gradient pattern of HC > PIGD > TD. Significant differences were detected only between HC and each PD subtype, while no statistically significant difference was observed between TD and PIGD. This reduced component value in TD patients may imply that tremor occurrence may be linked to attenuated VAN activation. As a key component of the attentional control network, VAN is responsible for attentional shifting, multimodal information integration and sensorimotor coordination. Existing studies have reported abnormal activation of the basal ganglia-thalamocortical circuit involving the sensorimotor cortex and attention networks in PD patients with tremor (25). The abnormal activation of the right angular gyrus in VAN observed in the present study may be associated with dysregulated automatic motor control and abnormal allocation of attentional resources related to tremor, offering new theoretical insights into the neural mechanisms of tremor.

#### 4.2.2 Subtype characteristics at the functional connectivity

Level Further analysis revealed that the FC strength between AN and SMN followed the trend of HC group >TD group >PIGD group, with the lowest connectivity detected in the PIGD group. This result was in accordance with more severe bradykinesia in PIGD patients. The AN-SMN FC mediates the integration of auditory input and motor output, and plays an essential role in speech-motor control, movement sequence execution and posture regulation. Prior studies have demonstrated that PIGD patients present a specific dynamic functional network pattern characterized by decoupling among the default mode network, AN and SMN. Moreover, the strength of AN-SMN connectivity is positively correlated with motor function scores (26, 27). The pronounced reduction of AN-SMN connectivity in the PIGD group may reflect more extensive impairment of neural integration, resulting in declined accuracy of postural gait control and speech-motor performance. Consistent with previous literature, this finding suggests that AN-SMN FC can be used to evaluate the severity of motor dysfunction in PIGD patients.

#### 4.2.3 Dynamic functional connectivity patterns

dFNC analysis showed that both PD subtypes had a lower occurrence rate of State 4 relative to the HC group, indicating impaired brain functional plasticity and synergistic activation capacity in all PD patients. This result aligns with the theory of compromised neural plasticity during PD progression. Previous research has confirmed that the temporal properties of dFNC states differ significantly between PD patients and HC. The number of brain network state transitions increases alongside cognitive deterioration, reflecting progressive decline of synergistic activation (28). Notably, no significant difference in this metric was observed between the two subtypes, indicating that reduced synergistic activation is a universal feature of PD rather than a subtype-specific alteration. This finding is consistent with the clinical manifestation that both subtypes suffer from functional deficits but differ in the types of impaired functions.

This study explored potential neuroimaging features derived from ICA-based dynamic functional connectivity and graph theory analysis to preliminarily characterize brain network alterations in PD patients and motor subtypes. Notably, most subtype-level differences observed in the present study were identified at the uncorrected statistical level and did not survive strict FDR correction. In addition, no significant pairwise differences were detected between the TD and PIGD subtypes for most network components and dynamic metrics. Therefore, all findings described in this section should be regarded as exploratory rather than validated diagnostic biomarkers. No imaging indicator identified in this work can currently support reliable clinical differentiation or definite subtype classification.

At the whole-group level, the decreased functional connectivity between the auditory network and sensorimotor network exhibited robust differences between PD patients and healthy controls after FDR correction, which may serve as a preliminary candidate feature reflecting impaired auditory-motor integration in PD. Similarly, the reduced occurrence rate of State 4 in dFNC analysis may tentatively reflect disrupted synergistic activation of multi-network coordination in PD, which could potentially indicate altered brain dynamic plasticity related to disease status.

At the subtype level, although several regional component values in the LFPN and RFPN showed trending differences between TD and PIGD patients, these discrepancies were not statistically rigorous after multiple comparison correction and lacked reliable pairwise subtype validation. Accordingly, these network alterations cannot be considered subtype-specific biomarkers. No stable tremor-related imaging feature was identified for TD subtype in the current cohort.

Collectively, the present neuroimaging results provide preliminary exploratory evidence for dynamic brain network abnormalities in PD and subtle subtype-associated functional alterations. However, given the limited sample size, cross-sectional design, uncorrected trending differences, and insufficient pairwise subtype contrasts, these features cannot yet be applied for clinical diagnosis or subtyping. Future large-sample, longitudinal studies are required to verify the stability, specificity, and translational value of these potential imaging indicators before clinical application.

## 5 Conclusion

In this study, we observed that PD patients (both TD and PIGD groups) exhibited spatial distribution abnormalities in multiple brain networks, including elevated component values of the right middle temporal gyrus in the AN and VAN, and of the right middle cingulate gyrus and right supplementary motor area in the SMN, together with decreased FC between the AN and SMN and a lower occurrence rate of State 4 in dFNC, relative to the HC group. The most consistent and robust finding across the study was therefore the divergence between PD patients and healthy individuals, supporting the view that a combination of ICA-derived dFNC and dynamic graph theory analysis can provide objective neuroimaging evidence for distinguishing PD patients from HC. With respect to the two motor subtypes (TD vs. PIGD), the between-group differences observed were more limited and less robust. We detected subtype-related differences in the component values of the left superior frontal gyrus within the LFPN and the right middle frontal gyrus within the RFPN. However, these subtype differences were not observed consistently across all analytical levels; for example, no significant difference was found between the two subtypes in the occurrence rate of State 4 in dFNC. Owing to the modest sample size and the cross-sectional design of this study, the present evidence is insufficient to conclusively establish subtype-specific neuroimaging markers. Accordingly, the capacity of these indicators to reliably differentiate the two motor subtypes should be regarded as preliminary, pending validation in larger, longitudinal cohorts.

In summary, the most solid conclusion drawn from the present study is the difference in dynamic brain network characteristics between PD patients and HC. The subtype-related alterations reported here should be interpreted with caution and warrant further investigation.

## Data Availability

The raw data supporting the conclusions of this article will be made available by the authors, without undue reservation.

## Footnotes

1
http://matlab.mrn.org/software/gift/

2
http://research.ics.tkk.fi/ica/icasso/

3
http://www.ibm.com/spss
